# A SCOPING REVIEW ON BARRIERS AND FACILITATORS OF COMMUNITY ENGAGEMENT IN LEARNING HEALTH SYSTEMS

**DOI:** 10.1093/geroni/igad104.3152

**Published:** 2023-12-21

**Authors:** Lillian Hung, Karen Lok Yi Wong, Annette Berndt, Tracy Windsor, Krisztina Vasarhelyi

**Affiliations:** University of British Columbia, Vancouver, British Columbia, Canada; University of British Columbia, Vancouver, British Columbia, Canada; University of British Columbia, Vancouver, British Columbia, Canada; University of British Columbia, Vancouver, British Columbia, Canada; Vancouver Coastal Health, Vancouver, British Columbia, Canada

## Abstract

This scoping review identifies the challenges and enablers of community engagement in Learning Health Systems (LHS), an area where elder and health care have growing interests on. LHS aims to improve healthcare delivery, translate knowledge into clinical practice, and emphasize patient-centred outcomes. We followed the Joanna Briggs Institute’s (JBI) scoping review methodology. First, keywords and index terms were identified from MEDLINE and CINAHL databases. Second, using the keywords and index terms identified in the first step, other databases like Web of Science, PsycINFO, and Google were searched. Third, the reference lists of the chosen literature were searched. The results include 25 papers. The Assessing Community Engagement (ACE) Conceptual Model by Aguilar-Gaxiola et al. was utilized to inform the analysis. Active community engagement enhances the relevancy and applicability of health research and fosters trust and rapport between healthcare providers and their communities. It is essential to have appropriate support, mutual respect, culturally sensitive and locally relevant processes with common-ground language. We identified several barriers to engagement: 1) meaningful motivations, 2) logistical challenges in processes, 3) disparities in power dynamics, 4) cultural misunderstanding, and 5) lack of knowledge and skills. The review underscores the need to move beyond tokenistic engagement towards genuine community collaborations. Future research should investigate the facilitators that enhance such meaningful partnerships.

